# Catalytic Hydrolysis of Paraoxon by Immobilized Copper(II) Complexes of 1,4,7-Triazacyclononane Derivatives

**DOI:** 10.3390/polym16202911

**Published:** 2024-10-16

**Authors:** Michaela Buziková, Hanna Zhukouskaya, Elena Tomšík, Miroslav Vetrík, Jan Kučka, Martin Hrubý, Jan Kotek

**Affiliations:** 1 Department of Inorganic Chemistry, Charles University, Hlavova 8, 128 40 Prague, Czech Republic; 2 Institute of Macromolecular Chemistry, Czech Academy of Sciences, Heyrovského náměstí 2, 162 06 Prague, Czech Republictomsik@imc.cas.cz (E.T.); jkucka@centrum.cz (J.K.); mhruby@centrum.cz (M.H.)

**Keywords:** paraoxon, nerve agents, macrocyclic copper complexes, thiophene polymers, catalysis, hydrolysis

## Abstract

Organophosphate neuroactive agents represent severe security threats in various scenarios, including military conflicts, terrorist activities and industrial accidents. Addressing these threats necessitates effective protective measures, with a focus on decontamination strategies. Adsorbents such as bentonite have been explored as a preliminary method for chemical warfare agent immobilization, albeit lacking chemical destruction capabilities. Chemical decontamination, on the other hand, involves converting these agents into non-toxic or less toxic forms. In this study, we investigated the hydrolytic activity of a Cu(II) complex, previously studied for phosphate ester hydrolysis, as a potential agent for chemical warfare decontamination. Specifically, we focused on a ligand featuring a thiophene anchor bound through an aliphatic spacer, which exhibited high hydrolytic activity in its Cu(II) complex form in our previous studies. Paraoxon, an efficient insecticide, was selected as a model substrate for hydrolytic studies due to its structural resemblance to specific chemical warfare agents and due to the presence of a chromogenic 4-nitrophenolate moiety. Our findings clearly show the hydrolytic activity of the studied Cu(II) complexes. Additionally, we demonstrate the immobilization of the studied complex onto a solid substrate of Amberlite XAD4 via copolymerization of its thiophene side group with dithiophene. The hydrolytic activity of the resultant material towards paraoxon was studied, indicating its potential utilization in organophosphate neuroactive agent decontamination under mild conditions and the key importance of surface adsorption of paraoxon on the polymer surface.

## 1. Introduction

CBRN (C—chemical, B—biological, R—radioactive and N—nuclear) agents pose a significant security threat in military conflicts, terrorist attacks and industrial or agrochemical accidents. Protection against them is therefore a key societal priority. The protection strategy involves many levels, ranging from organizational measures, monitoring and analytics to barrier protection, external decontamination means and antidotes for internal exposure. Therefore, the development of self-decontaminating materials capable of inactivating CBRN agents, whose decontamination effect is targeted at the surface of the skin; clothing; surfaces of articles of general use; or objects present in public space, including transport vehicles and other equipment, is of great importance.

**Figure 1:**
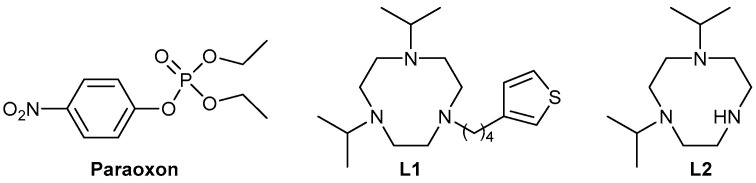
Structural formulas of compounds mentioned in the text.

In the case of chemical warfare agents (CWAs), their sorption onto a suitable material, e.g., layered silicate activated montmorillonite (bentonite, Desprach), can be used as a preliminary method but does not lead to chemical destruction of the CWA, only to its immobilization. Chemical decontamination of a CWA is carried out by reaction with suitable reagents that convert the CWA into non-toxic or less toxic products. Most commonly, mixtures containing “active chlorine”, most notably hypochlorite, oxidize a wide range of CWAs [1]; other (photo)oxidation processes are also studied [2]. Most CWAs are alkylating (yperite and its derivatives), acylating (phosgene or arsenic derivatives such as Lewisite) or phosphorylating (organophosphates) agents, and the strategy for their decomposition frequently involves the use of appropriate nucleophiles, such as thiosulfate, British anti-Lewisite, amidoximes/oximes, or water along with suitable catalysts or enzymes. However, each approach has its drawbacks [3]. Enzymatic hydrolysis is sometimes too substrate-specific, and the enzymes can be easily deactivated due to their lability and are often very expensive [4]. Furthermore, there is a problem with their immobilization onto a suitable solid support/material for easy handling and/or recovery [4,5,6]. To overcome this disadvantage, solid materials containing catalytic centers directly in their structure seem to be an alternative. Three-dimensional coordination polymer networks (metal-organic frameworks, “MOFs”) have shown fast degradation kinetics in some cases, especially with organophosphates [7,8,9], but they are often unstable in moist environments and complicated to handle [10,11]. Metal oxide nanoparticles usually perform poorly in protic solvents and water or need a high pH to reach a high conversion [12,13]. Catalysts on imprinted polymers are complex and expensive to produce and too specific to a single CWA [14]. For very hydrophobic CWAs, high catalytic efficiency can be reached by micellar catalysis [15]. On the other hand, for water-soluble materials, metal complexes working as artificial enzymes/hydrolases seem to be very potent [16]. Thanks to their small molecular weight, they can show very fast kinetics due to good substrate accessibility [17,18]. As artificial hydrolases, besides other transition metal ions, complexes especially of the biogenic Cu^2+^ or Zn^2+^ ions have often been utilized; the former benefits from its bioavailability, the latter from the general stability of its complexes due to the Irving–Williams order (although complexes of non-biogenic lanthanide ions have also been studied) [19,20,21,22,23]. However, the utilization of artificial hydrolases has mainly been focused on protein or nucleic acid hydrolysis [19,20,21]. Complexes used for these purposes must be coordinatively unsaturated to provide an easy approach to the substrate molecule. As a result, copper(II) complexes of a large family of ligands based on the 1,4,7-triazacyclononane scaffold were studied, as these complexes are—although coordinatively unsaturated—still, in general, very stable [24]. In such complexes, it is well known that the 1,4,7-triazacyclononane scaffold is facially coordinated to the central copper(II) ion, affording two coordination places for hydroxide/phosphate ligands [25,26,27]. In our previous contribution to this field [28], we studied a wide series of copper(II) complexes with ligands based on 1,4,7-triazacyclononane and their activity in catalytic hydrolysis of phosphate esters to obtain a deeper insight into the relationship between structure and properties. Some of our studied ligands were bifunctional, i.e., contained a side chain (linker) with a potentially anchoring group applicable for binding of the catalytic system onto a selected surface. Of those, ligand L1, with a thiophene anchor bound through an aliphatic spacer, showed the highest hydrolytic activity of its copper(II) complex (Figure 1). Therefore, we decided to explore its potential use in a relevant application. For this study, paraoxon (diethyl-4-nitrophenyl phosphate, Figure 1) was chosen as a model organophosphate, since paraoxon was widely used as an insecticide in the past [29,30] and has physicochemical properties close to those of some CWAs. Therefore, it is a compound of environmental interest [13]. Furthermore, it was used as a weapon of mass destruction (under the Project Coast chemical weapons program) during the apartheid era in South Africa [29]. Moreover, the hydrolysis of this substance is easily monitored spectrophotometrically, as it produces a chromogenic 4-nitrophenolate moiety.

In this manuscript, we present novel findings on the hydrolysis of paraoxon, focusing on the design and application of Cu(II)–L1 complexes. Initially, we confirm the anticipated hydrolytic activity of the Cu(II)–L1 complex and of a structurally related derivative, 1,4-diisopropyl-1,4,7-triazacyclononane (L2). The key innovation lies in the subsequent modification of a solid substrate through the immobilization of L1 via the oxidative polymerization of its thiophene side chain, resulting in poly(2,2′-dithiophene) integrated with the L1 ligand. This polymer was successfully deposited onto Amberlite resin microbeads, creating a high-surface-area catalyst. The coordination sites of L1 were saturated with Cu(II), and the hydrolytic activity of the resulting material against paraoxon was thoroughly investigated. This immobilization technique introduces significant practical advantages, such as easy removal from the reaction mixture by filtration and potential applications in environments requiring self-decontaminating surfaces.

## 2. Materials and Methods

### 2.1. General

Amberlite XAD4 20-60 mesh, 4-nitrophenyl phosphorodichloridate and 2,2′-dithiophene were purchased from Sigma-Aldrich (St. Louis, MO, USA). Ethanol (99.8%), formic acid and copper(II) sulfate pentahydrate were obtained from Lachner (Neratovice, Czech Republic). These and other commercial chemicals and solvents (Fluka (Buchs, Switzerland), Aldrich (St. Louis, MO, USA), CheMatech (Dijon, France), Lachema (Brno, Czech Republic)) were used as received. The ligands 1-(4-(thiophen-3-yl)butyl)-4,7-diisopropyl-1,4,7-triazonane (L1) and 1,4-diisopropyl-1,4,7-triazacyclononane (L2) were prepared using previously published procedures [28].

The ^1^H, ^31^P and ^13^C NMR spectra were acquired at 299 K with a Bruker Avance NEO 400 spectrometer operating at 400.1 MHz for ^1^H, 161.9 MHz for ^31^P and 100.6 MHz for ^13^C, using CDCl_3_ as a solvent. The morphology of prepared materials was analyzed by scanning electron microscopy (SEM) using a MAIA3 microscope (TESCAN, Brno, Czech Republic). Copper content in the prepared Amberlite-adsorbed material was determined by atomic absorption spectroscopy (AAS). A sample of the analyzed material (10–20 mg) was mixed with 2 ml of conc. HNO_3_, and the mixture was evaporated in a fume hood at 100 °C. This process was repeated one more time. The solid residue was extracted with diluted aq. HNO_3_ (2%), and the remaining Amberlite beads were removed by filtration through a PVDF microfilter. The extract was subjected to AAS analysis.

The molecular structure of the polymeric materials was studied using a inVia Qontor Raman spectrometer (Renishaw, New Mills, UK) equipped with an 830 nm laser and a holographic grating density of 2400 lines mm^−1^. A research-grade DM 2700M microscope (Leica Microsystems, Wetzlar, Germany)with an LWD Leica objective lens was used to focus the laser beam onto the sample. A Renishaw Centrus CCD detector was used to record the spectra.

Fourier-transform infrared (FTIR) spectra were recorded at room temperature on a Thermo Fisher Scientific Nicolet iS50 FTIR spectrometer (4 cm^−1^ resolution, Happ–Genzel apodization) in the 400–4000 cm^−1^ (KBr beam splitter) region using attenuated total reflectance (ATR) technique on the diamond crystal. Standard ATR correction (Thermo Nicolet Omnic 9.2 software [31]) was applied to the recorded spectra.

X-ray photoelectron spectroscopy (XPS) measurements were performed utilizing a K-Alpha+ spectrometer from ThermoFisher Scientific, located in East Grinstead, UK. The samples underwent analysis using a micro-focused, monochromated Al Kα X-ray source, possessing a spot size of 400 μm. The angle of incidence was set at 30° relative to the surface, while the emission angle was perpendicular to the surface.

### 2.2. Synthesis

#### 2.2.1. Paraoxon

4-nitrophenyl phosphorodichloridate (7.50 g, 29.3 mmol) was slowly added to anhydrous ethanol (50 mL) in a 100 mL round-bottom flask cooled in an ice bath with stirring. Triethylamine (9 mL, 64.6 mmol) was slowly added through a septum, and after the last portion, the mixture was left to warm to room temperature and was then stirred for the next 2 h. The resulting mixture was concentrated in vacuo using a rotary evaporator, and the residue was fractionated between water and diethyl ether. The organic layer was separated and concentrated using a rotary evaporator. As the residue still contained some triethylamine hydrochloride, the extraction step was repeated one more time. The diethyl ether layer was dried with anhydrous magnesium sulfate and evaporated, yielding 7.99 g (99%) of the product. The purity was verified by thin-layer chromatography (TLC, Silica gel 60 F254), and the *R*_f_ of the product was 0.7 in diethyl ether as the mobile phase.

NMR (*δ*, ppm): ^1^H: 8.17 (d, 2H, Ar), 7.32 (d, 2H, Ar), 4.18 (p, 4H, CH_2_), 1.30 (t, 6H, CH_3_); ^31^P{^1^H}: −7.30; ^13^C: 155.64, 144.69, 125.72, 120.60, 65.22, 16.10.

#### 2.2.2. Polymer Materials

Wet commercial Amberlite XAD4 (20–60 mesh) was thoroughly washed with water to remove preservatives (NaCl and Na_2_CO_3_) and the washed beads were dried in vacuo. Dry beads (330 mg) were dispersed in 4.7 mL of formic acid, and 2,2′-dithiophene (56 mg, 0.34 mmol) dissolved in 1 mL of EtOH was added, followed by the addition of a solution of L1 (50 mg, trifluoroacetate salt, 0.07 mmol) in 1 mL of EtOH. To the mixture, a solution of (NH_4_)_2_S_2_O_8_ (15.2 mg, 0.12 mmol) in 1 mL of water was added, and the resulting reaction mixture was stirred at 70 °C in a sealed ampule for 24 h, during which it turned dark blue. The residue was diluted with water (10 mL), and the modified beads were separated by centrifugation followed by repeated washing with water until the supernatant was colorless.

A portion of the modified Amberlite beads (ca. 2/3) was mixed with 5 mL of 0.1 M aqueous solution of CuSO_4_ and stirred for 24 h at room temperature. The copper-loaded beads were thoroughly washed with water for the next 24 h, separated by centrifugation and dried under vacuum. AAS analysis revealed a copper content of 0.10% in the dried solid material.

The sample of 2,2′-dithiophene–L1 copolymer for spectral characterization was prepared in the same way as described above, but the reaction proceeded in the absence of the Amberlite beads. The polymer was isolated by centrifugation of the reaction mixture and was thoroughly washed with water, ethanol and acetone and dried in vacuo, affording black material, somewhat soluble in dichloromethane, chloroform and dimethylsulfoxide (DMSO), forming very dark blue–green to black solutions.

A sample of pristine polythiophene was obtained by polymerization of 2,2′-dithiophene performed under the same conditions as described above and was isolated by precipitation on addition of water. The black material was filtered, thoroughly washed with water and dried in vacuo.

#### 2.2.3. Kinetic Measurements

Paraoxon stock solution (10 mM) was prepared by dissolution of pure liquid paraoxon in water. Stock solutions of Cu(II)–L complexes (0.10 M) were prepared by mixing the appropriate amount of solid CuSO_4_∙5H_2_O with aq. solution of the ligand in slight excess (10% mol.), pH adjustment to 7.4 with NaOH and dilution to the required volume. Buffer solutions (0.30 M) were prepared from solid 2-(*N*-morpholino)ethanesulfonic acid (MES) or tris(hydroxymethyl)aminomethane (TRIS) and their pH was adjusted to 6.5 (MES) or 7.4 (TRIS) with aq. NaOH/HCl.

For kinetic experiments, buffered reaction mixtures (0.15 M buffer; pH 6.5 MES, pH 7.4 TRIS) containing 1.0 mM paraoxon and 10 mM CuSO_4_ or the respective Cu(II)–L complex were prepared by mixing appropriate amounts of paraoxon (10 mM) and CuSO_4_ or Cu(II)–L (0.10 M) stock solutions with the buffer stock solution (0.30 M) and water. Reaction vessels were tightly closed and stirred at ambient temperature (room temperature controlled at 23 °C) or in an oil bath at the chosen elevated temperature (37 or 50 °C). At chosen times covering up to 6 days, small amounts of the reaction mixture (three samples of 100 μL; all measurements were performed in triplicate) were pipetted into the wells of a microplate. The absorbance of the samples was measured at 400 nm on a Varioscan LUX Multimode Microplate Reader (Thermo Fisher Scientific; Waltham, MA, USA) at ambient temperature; for the choice of wavelength, see Figure S1. The blank sample consisted of pure buffer (measurement of spontaneous hydrolysis), buffer with 10 mM CuSO_4_ (measurement of influence of free Cu(II) ions) or buffer with the respective Cu(II)–L complex (10 mM, measurement of catalytic efficiency of the complexes).

Using calibration curves of 4-nitrophenol (range of 0.001–0.04 mM taken in the respective buffer), concentrations of 4-nitrophenolate were calculated and were used for calculation of 4-nitrophenol and paraoxon concentrations. The expected pseudo-first-order kinetics of the hydrolytic reaction was confirmed by the linear dependence of the semilogarithmic plot ln(*c*_0_/*c*) vs. time [*c*_0_ is the initial concentration of paraoxon (1 mM), and *c* is its concentration at a given time point]. Observed rate constants *k*_obs_ were determined from the time dependence of measured absorbance by linear (in most cases) or exponential fitting of the data. Plots are shown in ESI (Figures S2–S7) with the respective fitting parameters. The pseudo-first-order kinetics was also confirmed by performing the hydrolytic reaction in a 3:1 ethanol/water mixture (Figure S8), where the observed rate constant was approx. 4 times lower than that of the reaction performed in water due to lower water accessibility in such a medium.

Hydrolytic experiments employing solid material catalysts were performed and followed analogously as described above, starting from paraoxon solution (1 mM) in TRIS buffer (0.15 M, pH = 7.4, 1.0 ml) to which the studied solid material (native Amberlite, Amberlite covered with metal-free 2,2′-dithiophene–L1 copolymer and Amberlite with 2,2′-dithiophene–L1 copolymer loaded with the Cu(II) ions; 30 mg of the dried form) was added, and the reaction mixture was stirred in an oil bath with a controlled temperature of 50 °C. Due to the beads partially mechanically disintegrating while stirring, we were unable to conduct these kinetic experiments for a period longer than 2 days. After 2 days, the mixtures became cloudy, and the increased turbidity affected the optical absorbance of the solutions (Figure S9).

## 3. Results and Discussion

### 3.1. Synthesis of Polymeric Material

**Scheme 1:**
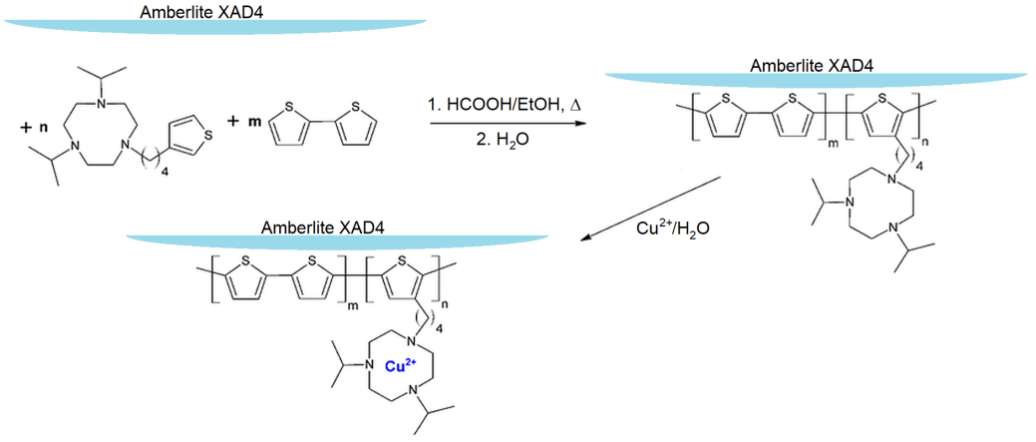
Preparation of the polymer catalyst by statistical copolymerization of 2,2′-dithiophene with ligand L1. Noncovalent (hydrophobic) binding/sorption of the polymer on the Amberlite XAD4 beads surface is expected. Macroporous Amberlite XAD4 beads consist of crosslinked polystyrene.

Ligand L1 bears a thiophene side group allowing a simple incorporation into a polythiophene polymer. As a solid support of the resulting polymer, Amberlite spherical microbeads were chosen. Amberlite is a macroporous resin, prepared by copolymerization of styrene and divinylbenzene. Due to its high chemical and physical stability, which allows its use at different pH and high temperatures, it is often used as a carrier of active substances in a variety of fields, including toxicology, medicine, ecology and the food industry [32,33,34,35,36]. Amberlite has a non-ionic and strictly hydrophobic character, and its coverage by hydrophobic polythiophene is expected, according to Scheme 1.

To introduce metal-chelating groups on the surface of the Amberlite beads, statistical radical copolymerization [37] of 2,2′-dithiophene with ligand L1 initiated by peroxodisulfate was performed in the presence of the beads in a mixture of EtOH and formic acid with heating under the ambient atmosphere. The reaction proceeded for several days as evidenced by a color change to deep blue, which is typical for polythiophenes. After the reaction, the mixture was diluted with water, and the dark blue beads were centrifuged and thoroughly washed with water.

The binding/adsorption of the deep blue thiophene polymer onto the polymeric Amberlite support by non-covalent strong hydrophobic and π–π (conjugated thiophene-based polymer–aromatic styrene monomeric unit in Amberlite resin) interactions was stable as demonstrated by a lack of color leakage from the solid material in aqueous suspension. Unfortunately, this strong adsorption also prevented thorough characterization of the polymeric material.

Mixing with a solution of CuSO_4_ and washing the copper(II)-loaded beads afforded the material used in catalytic experiments. The copper content in the dried material was 0.10%.

Scanning electron microscopy revealed that neither the surface modification of the Amberlite beads with polythiophene nor the copper(II) loading of the material affected the surface morphology of the beads (Figure S10).

However, the adsorbed 2,2′-dithiophene–L1 copolymer is only a tiny part of the solid Amberlite-supported material and cannot be non-destructively removed from the surface and further characterized. Therefore, we performed the polymerization reaction under the same conditions but in absence of the Amberlite beads to obtain (non-adsorbed) 2,2′-dithiophene–L1 copolymer, which was further studied spectroscopically. For the characterization, we used NMR, Raman, infrared and X-ray photoelectron spectroscopies. Pristine poly-2,2′-dithiophene (formally polythiophene) was also prepared for comparison.

Although the prepared polymers seem to be at least partly soluble in chlorinated solvents and DMSO and very dark blue–green (almost black) samples were obtained, no significant signals were found in the NMR spectra of either the pristine poly-2,2′-dithiophene or the 2,2′-dithiophene–L1 copolymer samples dissolved in CDCl_3_ or DMSO-d6. Such behavior can be attributed to presence of radicals stabilized by the conductive nature of the polythiophene backbone. This explanation is supported by a color shift of the polymer solutions from blue to green when the materials were manipulated in air during the workup. The stabilization of cation radicals in the oxidized polythiophene backbone is well known [38]. In this case, the radicals present in the sample effectively relax the NMR signals, and it cannot be reliably measured.

Therefore, we used Raman and IR spectroscopies to study the structure of the polymers and confirm a presence of the aliphatic CH_2_/CH_3_ groups in the studied copolymer. Pristine poly-2,2′-dithiophene was measured for comparison. The full Raman spectra in the region from 200–3200 cm^−1^ and FTIR ATR spectra in the region 400–4000 cm^−1^ are shown in Figures S11 and S12, respectively. In the Raman spectrum, we are particularly interested in the region 1250–1650 cm^−1^, because it includes the two main in-plane ring modes: the C=C symmetric stretching at 1445 cm^−1^ [38,39] and the C–C intra-ring stretching at 1380 cm^−1^, which is known to be sensitive to π-electron delocalization [40] and degree of structural order in poly(thiophenes) [39,41]. The pristine poly-2,2′-dithiophene has narrow peaks compared to the 2,2′-dithiophene–L1 copolymer. The broadening of the peak for copolymer indicates some out-of-plane disorder in its structure, which can be explained by the presence of the side chain with 1,4,7-triazacyclononane moieties. In addition, the Raman spectrum of the copolymer showed stronger photoluminescence than pristine poly-2,2′-dithiophene, which complicates the observation of weak Raman bands. However, in the FTIR ATR spectra of the copolymer, a distinct band in region 2850–2880 cm^−1^ typical for aliphatic C–H groups was observed, confirming the presence of L1 (as it is the only possible source of such groups) in the copolymer. Incorporation of L1 in the copolymer was confirmed beyond doubt by the XPS measurement, as the presence of nitrogen (although at low level) was clearly found only in the 2,2′-dithiophene–L1 copolymer, whereas no nitrogen was found in the sample of pristine poly-2,2′-dithiophene (Figure S13 and Table S1).

### 3.2. Paraoxon Hydrolysis

**Scheme 2:**
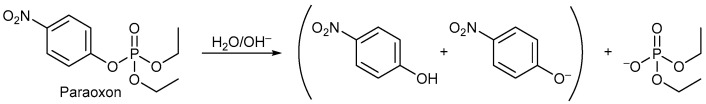
Hydrolysis of paraoxon.

The hydrolysis of paraoxon produces a molecule of 4-nitrophenol (or 4-nitrophenolate anion, depending on pH; Scheme 2).

To find suitable parameters for the UV-Vis spectral measurements, we acquired the spectra of paraoxon and 4-nitrophenol under different conditions (Figure S1). The absorption band of paraoxon (*λ*_max_ = 275 nm) slightly overlaps with the absorption band of 4-nitrophenol (*λ*_max_ = 317 nm), but both compounds are well distinguished from the absorption band of 4-nitrophenolate anion (*λ*_max_ = 400 nm). Therefore, the kinetic experiments were conducted at 400 nm. As the p*K*_A_ of 4-nitrophenol is 7.15, the 4-nitrophenolate anion is not significantly generated at pH < 6. Therefore, pH 6.5 and 7.4 (buffered with MES for pH 6.5 and TRIS for pH 7.4) were chosen for the kinetic experiments, as a significant band for the 4-nitrophenolate anion appears under these conditions.

Pseudo-first-order kinetics of the hydrolytic reaction was expected, as the concentration of water is much higher than that of paraoxon and can be assumed to be constant during the reaction. This was confirmed by the linear dependence of the semilogarithmic plot ln(*c*_0_/*c*) vs. time (see Experimental). Furthermore, for the Cu–L2 complex (chosen for comparison as it showed very high efficiency in our previous study) [28], we performed the hydrolytic reaction in both the aqueous solution and the 3:1 ethanol/water mixture. In these experiments, the observed rate constants *k*_obs_ obtained in the aqueous solution dropped approx. fourfold when performing the reaction in the 3:1 ethanol/water mixture due to approx. fourfold lower water accessibility (under the pseudo-first-order conditions, the water concentration is included in the apparent rate constant); see Table 1 and Figures S7 and S8. Thus, the apparent rate constants *k*_obs_ could be determined as the slopes of the initial linear part of the time dependence of absorbance at 400 nm (in most cases) or by exponential fitting (in the experiment with Cu–L1 performed at 50 °C, where the absorbance already clearly had exponential dependence due to the high hydrolytic activity of this complex at this temperature).

As paraoxon can undergo slow spontaneous hydrolysis or its hydrolysis can be potentially influenced by the presence of free metal ions, we evaluated the hydrolysis of paraoxon in pure buffers and then in the presence of CuSO_4_. The influence of CuSO_4_ on the hydrolytic reaction could be studied only in TRIS buffer (pH 7.4), as it is slightly complexing, and no precipitation of Cu(OH)_2_ occurred even at this pH; however, a precipitate was formed at pH 6.5 when MES buffer was employed. The fits of kinetic data are shown in Figures S2–S4; the observed apparent rate constants of the hydrolytic reaction are listed in Table 2 and the calculated half-times in Table 3. Hydrolysis of paraoxon is very slow, with a half-time on the order of thousands of hours, and the presence of “free” Cu(II) has no influence on the hydrolysis of paraoxon (although it seems that it even has a “positive” effect on paraoxon’s stability, the calculated rate constants and half-times are loaded by a high error, as the reaction is extremely slow and reaches conversion of only several % after 6 days). On the contrary, if Cu–L1 or Cu–L2 was present in the reaction mixture, the hydrolysis proceeded much faster (Table 2 and Table 3, Figures S5–S7). Under the conditions chosen, the reaction rate was increased by a factor of up to 135 in the presence of Cu–L2 and even 210 in the presence of Cu–L1, when compared with the spontaneous hydrolysis at pH 7.4. Unfortunately, data for the Cu–L1 complex at pH 6.5 could not be acquired due to the appearance of a colloidal precipitate.

Given the promising results shown above, we performed a hydrolytic reaction with the prepared polymeric materials (i.e., under heterogeneous conditions). The reactions were performed at pH 7.4 and 50 °C. Besides the Cu(II)-loaded material, we also tested a hydrolytic reaction in the presence of unmodified Amberlite beads and beads modified with 2,2′-dithiophene–L1 copolymer. Plots of these kinetic reactions are shown in Figure S9, and apparent rate constants and half-times are listed in Table 4.

Although the significant slowdown of hydrolysis caused by the heterogeneous nature of the polymer catalyst when compared to homogenous conditions was observed, there was a clear acceleration of hydrolysis after the complexation of the copper(II) ions into the immobilized ligand Amberlite–2,2′-dithiophene–L1 compared to the immobilized ligand Amberlite–2,2′-dithiophene–L1 (by a factor of ca 2.5–3), most plausibly due to the catalytic effect of the copper(II) complex as anticipated. The apparent hydrolysis rate on the beads is, in general, slowed down, probably due to efficient hydrophobic adsorption of paraoxon (lowering paraoxon mobility) and/or nitrophenol as a hydrolysis product (preventing its detection in the solution) onto the hydrophobic surface of the beads. This also explains why the apparent hydrolysis rate on beads is lower than the spontaneous hydrolysis rate in a buffer at the same temperature. Although this may slow down the apparent hydrolysis rate, it also means that a significantly lower quantity of toxic species remains in the solution and that the self-decontamination of the adsorbed paraoxon on the beads’ surface is slow but ongoing.

## 4. Conclusions

In conclusion, our study presents promising results regarding the potential use of a copper(II) complex featuring a ligand with a thiophene anchor bound through an aliphatic spacer as a catalyst for CBRN decontamination. In hydrolytic studies using paraoxon as a model organophosphate, we confirmed the expected high hydrolytic activity of the Cu(II) complex, indicating its efficacy in breaking down toxic compounds. The immobilization of the studied complex onto a solid substrate, specifically Amberlite XAD4, via copolymerization of its thiophene side arm further enhances its utility by enabling facile catalyst removal from reaction mixtures and offering opportunities for surface adsorption of paraoxon. Further research in this direction may yield advancements in materials and methodologies for mitigating the risks posed by CBRN agents.

## Figures and Tables

**Table 1:** Comparison of observed (apparent) rate constants *k*_obs_ (s^−1^) and half-times (h) for hydrolysis of paraoxon in the presence of the Cu(II)–L2 complex in various solvents. *c*(Paraoxon) = 1.0 mM, *c*(Cu–L2) = 10 mM, *c*(TRIS) = 0.15 M; pH = 7.4; *t* = 23, 37 or 50 °C.

	Buffer (pH 7.4)			3:1 (*v*:*v*) Ethanol/Buffer (pH 7.4)		
parameter	23 °C	37 °C	50 °C	23 °C	37 °C	50 °C
*k*_obs_ (s^−1^)	1.35∙10^−5^	4.79∙10^−5^	1.30∙10^−4^	4.70·10^−6^	1.33·10^−5^	2.93·10^−5^
half-time (h)	14	4.0	1.5	41	14.5	6.6

**Table 2:** Observed (apparent) rate constants *k*_obs_ (s^−1^) for hydrolysis of paraoxon under different conditions. *c*(Paraoxon) = 1.0 mM, *c*(CuSO_4_) = 10 mM, *c*(Cu–L) = 10 mM, *c*(buffer) = 0.15 M; pH = 6.5 (MES) or 7.4 (TRIS); *t* = 23, 37 or 50 °C.

	pH = 6.5			pH = 7.4		
catalyst	23 °C	37 °C	50 °C	23 °C	37 °C	50 °C
none ^1^	9.26·10^−8^	1.60·10^−7^	2.59·10^−7^	3.59·10^−7^	4.48·10^−7^	9.61·10^−7^
CuSO_4_	pptn. ^2^	pptn. ^2^	pptn. ^2^	1.49·10^−7^	3.67·10^−7^	7.67·10^−7^
Cu–L1	pptn. ^2^	pptn. ^2^	pptn. ^2^	1.02·10^−5^	1.74·10^−5^	2.02∙10^−4^
Cu–L2	2.99·10^−5^	3.83·10^−5^	7.85·10^−5^	1.35·10^−5^	4.79·10^−5^	1.30·10^−4^

^1^ Spontaneous (no catalyst added). ^2^ Precipitation occurred.

**Table 3:** Half-times (h) for hydrolysis of paraoxon under different conditions. *c*(Paraoxon) = 1.0 mM, *c*(CuSO_4_) = 10 mM, *c*(Cu–L) = 10 mM, *c*(buffer) = 0.15 M; pH = 6.5 (MES) or 7.4 (TRIS); *t* = 23, 37 or 50 °C.

	pH = 6.5			pH = 7.4		
catalyst	23 °C	37 °C	50 °C	23 °C	37 °C	50 °C
none ^1^	2080	1201	742	537	430	200
CuSO_4_	pptn. ^2^	pptn. ^2^	pptn. ^2^	1290	525	251
Cu–L1	pptn. ^2^	pptn. ^2^	pptn. ^2^	19	11	1.0
Cu–L2	6.5	5.0	2.5	14	4.0	1.5

^1^ Spontaneous (no catalyst added). ^2^ Precipitation occurred.

**Table 4:** Observed (apparent) rate constants *k*_obs_ (s^−1^) and half-times (h) for hydrolysis of paraoxon in the presence of Amberlite beads modified with 2,2′-dithiophene–L1 copolymer loaded with Cu(II) and those of the parent materials used for comparison. pH = 7.4 (0.15 M TRIS), 50 °C.

Material	*k*_obs_ (s^−1^)	*t*_½_ (h)
native Amberlite	3.60∙10^−7^	534
Amberlite–2,2′-dithiophene–L1	2.42∙10^−7^	796
Amberlite–2,2′-dithiophene–L1–Cu(II)	6.65∙10^−7^	290

## Data Availability

The raw/processed data required to reproduce these findings cannot be shared at this time due to technical limitations but are available on request.

## Supplementary Materials

The following supporting information can be downloaded at: https://www.mdpi.com/article/10.3390/polym16202911/s1, Figure S1: UV-Vis spectra of 4-nitrophenol in selected buffers at various pH values; Figures S2 and S3: First-order kinetic plots of spontaneous (uncatalyzed) paraoxon hydrolysis at various temperatures and pH values; Figure S4: First-order kinetic plots of paraoxon hydrolysis in the presence of CuSO_4_ at various temperatures; Figures S5–S9: First-order kinetic plots of paraoxon hydrolysis in the presence of studied complexes and polymers at various temperatures and pH values; Figure S10: Morphology of materials studied by scanning electron microscopy; Figure S11: Raman spectra of pristine poly-2,2′-dithiophene and 2,2′-dithiophene–L1 copolymer; Figure S12: FTIR ATR spectrum of the 2,2′-dithiophene–L1 copolymer; Figure S13: Graphical representations of the results of XPS measurements of pristine poly-2,2′-dithiophene and 2,2′-dithiophene–L1 copolymer; Table S1: Compilation of XPS results (Atomic %) obtained for pristine poly-2,2′-dithiophene and 2,2′-dithiophene–L1 copolymer.

## References

[1] Bjarnason S., Mikler J., Hill I., Tenn C., Garrett M., Caddy N., Sawyer T.W. (2008). Comparison of selected skin decontaminant products and regimens against VX in domestic swine. Hum. Exp. Toxicol..

[2] Silva M., Baltrusaitis J. (2021). Destruction of emerging organophosphate contaminants in wastewater using the heterogeneous iron-based photo-Fenton-like process. J. Hazard. Mater. Lett..

[3] Janoš P., Tokar O., Došek M., Mazanec K., Ryšánek P., Kormunda M., Henych J., Janoš P. (2021). Amidoxime-functionalized bead cellulose for the decomposition of highly toxic organophosphates. RSC Adv..

[4] Thakur M., Medintz I.L., Walper S.A. (2019). Enzymatic Bioremediation of Organophosphate Compounds–Progress and Remaining Challenges. Front. Bioeng. Biotechnol..

[5] Wang L., Sun Y. (2021). Engineering organophosphate hydrolase for enhanced biocatalytic performance: A review. Biochem. Eng. J..

[6] Lou Y., Zhang B., Ye X., Wang Z.-G. (2023). Self-assembly of the de novo designed peptides to produce supramolecular catalysts with built-in enzyme-like active sites: A review of structureeactivity relationship. Mater. Today Nano.

[7] Li S., Zhou Z., Tie Z., Wang B., Ye M., Du L., Cui R., Liu W., Wan C., Liu Q. (2022). Data-informed discovery of hydrolytic nanozymes. Nat. Commun..

[8] Moon S.-Y., Liu Y., Hupp J.T., Farha O.K. (2015). Instantaneous hydrolysis of nerve-agent simulants with a six-connected zirconium-based metal–organic framework. Angew. Chem. Int. Ed..

[9] Islamoglu T., Ortuno M.A., Proussaloglou E., Howarth A.J., Vermeulen N.A., Atilgan A., Asiri A.M., Cramer C.J., Farha O.K. (2018). Presence versus Proximity: The Role of Pendant Amines in the Catalytic Hydrolysis of a Nerve Agent Simulant. Angew. Chem. Int. Ed..

[10] DeCoste J.B., Peterson G.W., Schindler B.J., Killops K.L., Browe M.A., Mahle J.J. (2013). The effect of water adsorption on the structure of the carboxylate containing metal–organic frameworks Cu-BTC, Mg-MOF-74, and UiO-66. J. Mater. Chem. A.

[11] DeCoste J.B., Peterson G.W. (2014). Metal–Organic Frameworks for Air Purification of Toxic Chemicals. Chem. Rev..

[12] Denet E., Espina-Benitez M.B., Pitault I., Pollet T., Blaha D., Bolzinger M.-A., Rodriguez-Nava V., Briançon S. (2020). Metal oxide nanoparticles for the decontamination of toxic chemical and biological compounds. Int. J. Pharm..

[13] Zhan S.-W., Tseng W.-B., Tseng W.-L. (2020). Impact of nanoceria shape on degradation of diethyl paraoxon: Synthesis, catalytic mechanism, and water remediation application. Environ. Res..

[14] Pan J., Liu S., Jia H., Yang J., Qin M., Zhou T., Chen Z., Jia X., Guo T. (2019). Rapid hydrolysis of nerve agent simulants by molecularly imprinted porous crosslinked polymer incorporating mononuclear zinc(II)-picolinamine-amidoxime module. J. Catal..

[15] Cabal J., Míčová J., Kuča K. (2010). Kinetics of hydrolysis of organophosphate soman by cationic surfactant Resamin AE. J. Appl. Biomed..

[16] Zhang B., Breslow R. (1997). Ester Hydrolysis by a Catalytic Cyclodextrin Dimer Enzyme Mimic with a Metallobipyridyl Linking Group. J. Am. Chem. Soc..

[17] Feng G., Natale D., Prabaharan R., Mareque-Rivas J.C., Williams N.H. (2006). Efficient Phosphodiester Binding and Cleavage by a Zn^II^ Complex Combining Hydrogen-Bonding Interactions and Double Lewis Acid Activation. Angew. Chem. Int. Ed..

[18] Liu C., Wang L. (2009). DNA hydrolytic cleavage catalyzed by synthetic multinuclear metallonucleases. Dalton Trans..

[19] Desbouis D., Troitsky I.P., Belousoff M.J., Spiccia L., Graham B. (2012). Copper(II), zinc(II) and nickel(II) complexes as nuclease mimetics. Coord. Chem. Rev..

[20] Wende C., Lüdtke C., Kulak N. (2014). Copper Complexes of N-Donor Ligands as Artificial Nucleases. Eur. J. Inorg. Chem..

[21] Li F.-Z., Feng F.-M., Yu L., Xie J.-Q. (2014). Nucleic acid and phosphoester hydrolytic cleavage catalysed by aza-crown ether metal complexes as synthetic nucleases. Prog. React. Kin. Mech..

[22] Yu L., Li F.-Z., Wu J.-Y., Xie J.-Q., Li S. (2016). Development of the aza-crown ether metal complexes as artificial hydrolase. J. Inorg. Biochem..

[23] Tomczyk M.D., Kuźnik N., Walczak K. (2023). Cyclen-based artificial nucleases: Three decades of development (1989–2022). Part a–Hydrolysis of phosphate esters. Coord. Chem. Rev..

[24] Martell A.E., Smith R.M., Motekaitis R.J. (2003). NIST Standard Reference Database 46 (Critically Selected Stability Constants of Metal Complexes).

[25] Deal K.A., Burstyn J.N. (1996). Mechanistic studies of dichloro(1,4,7-triazacyclononane)copper(II)-catalyzed phosphate diester hydrolysis. Inorg. Chem..

[26] Deck K.M., Tseng T.A., Burstyn J.N. (2002). Triisopropyltriazacyclononane copper(II): An efficient phosphodiester hydrolysis catalyst and DNA cleavage agent. Inorg. Chem..

[27] Fry F.H., Fischmann A.J., Belousoff M.J., Spiccia L., Brügger J. (2005). Kinetics and mechanism of hydrolysis of a model phosphate diester by [Cu(Me_3_tacn)(OH_2_)_2_]^2+^ (Me_3_tacn = 1,4,7-trimethyl-1,4,7-triazacyclononane). Inorg. Chem..

[28] Buziková M., Willimetz R., Kotek J. (2023). The Hydrolytic Activity of Copper(II) Complexes with 1,4,7-Triazacyclononane Derivatives for the Hydrolysis of Phosphate Diesters. Molecules.

[29] Gupta R.C. (2011). Toxicology of Organophosphate and Carbamate Compounds.

[30] Lorke D.E., Nurulain S.M., Hasan M.Y., Kuča K., Petroianu G.A. (2020). Combined Pre- and Posttreatment of Paraoxon Exposure. Molecules.

[31] Omnic 9.2.98.

[32] Shanbhag M.M., Ilager D., Mahapatra S., Shetti N.P., Chandra P. (2021). Amberlite XAD-4 based electrochemical sensor for diclofenac detection in urine and commercial tablets. Mater. Chem. Phys..

[33] Kara D., Fisher A., Hill S.J. (2009). Determination of trace heavy metals in soil and sediments by atomic spectrometry following preconcentration with Schiff bases on Amberlite XAD-4. J. Hazard. Mater..

[34] Solangi I.B., Memon S., Bhanger M.I. (2009). Removal of fluoride from aqueous environment by modified Amberlite resin. J. Hazard. Mater..

[35] Kyriakopoulos G., Doulia D., Anagnostopoulos E. (2005). Adsorption of pesticides on porous polymeric adsorbents. Chem. Eng. Sci..

[36] Ahmad A., Siddique J.A., Laskar M.A., Kumar R., Mohd-Setapar S.H., Khatoon A., Shiekh R.A. (2015). New generation Amberlite XAD resin for the removal of metal ions: A review. J. Environmen. Sci..

[37] Ji H., Zhang X., Zhang X.-H., Theato P. (2021). Chapter 8: Thiophene-based polymers: Synthesis and applications. Sulfur-Containing Polymers: From Synthesis to Functional Materials.

[38] Brambilla L., Capel Ferrón C., Tommasini M., Hong K., López Navarrete J.T., Hernández V., Zerbi G. (2018). Infrared and multi-wavelength Raman spectroscopy of regio-regular P3HT and its deutero derivatives. J. Raman Spectrosc..

[39] Paternò G.M., Robbiano V., Fraser K.J., Frost C., García Sakai V., Cacialli F. (2017). Neutron Radiation Tolerance of Two Benchmark Thiophene-Based Conjugated Polymers: The Importance of Crystallinity for Organic Avionics. Sci. Rep..

[40] Gao Y., Grey J.K. (2009). Resonance chemical imaging of polythiophene/fullerene photovoltaic thin films: Mapping morphology-dependent aggregated and unaggregated C=C Species. J. Am. Chem. Soc..

[41] Tsoi W.C., James D.T., Kim J.S., Nicholson P.G., Murphy C.E., Bradley D.D.C., Nelson J., Kim J.-S. (2011). The nature of in-plane skeleton Raman modes of P3HT and their correlation to the degree of molecular order in P3HT:PCBM blend thin films. J. Am. Chem. Soc..

