# Supplementary material for: Catalytic Hydrolysis of Paraoxon by Immobilized Copper(II) Complexes of 1,4,7-Triazacyclononane Derivatives
Source: Polymers (Basel). 2024 Oct 16;16(20):2911. doi: 10.3390/polym16202911 (PMC11510994; doi:10.3390/polym16202911)
Supplement: Supplementary file 1 [file polymers-16-02911-s001.zip › polymers-3164931-supplementary.pdf]

Electronic Supporting Information

for

Catalytic Hydrolysis of Paraoxon by Immobilized Copper(II) Complexes of 1,4,7-Triazacyclononane Derivatives

Michaela Buziková [1], Hanna Zhukouskaya [2], Elena Tomšík [2], Miroslav Vetrík [2], Jan Kučka [2], Martin Hrubý [2] and Jan Kotek [1],*

1 Department of Inorganic Chemistry, Charles University, Hlavova 8, 128 40 Prague, Czech Republic

2 Institute of Macromolecular Chemistry, Czech Academy of Sciences, Heyrovského náměstí 2, 162 06 Prague, Czech Republic;

tomsik@imc.cas.cz (E.T.); jkucka@centrum.cz (J.K.); mhruby@centrum.cz (M.H.)
* Correspondence: modrej@natur.cuni.cz

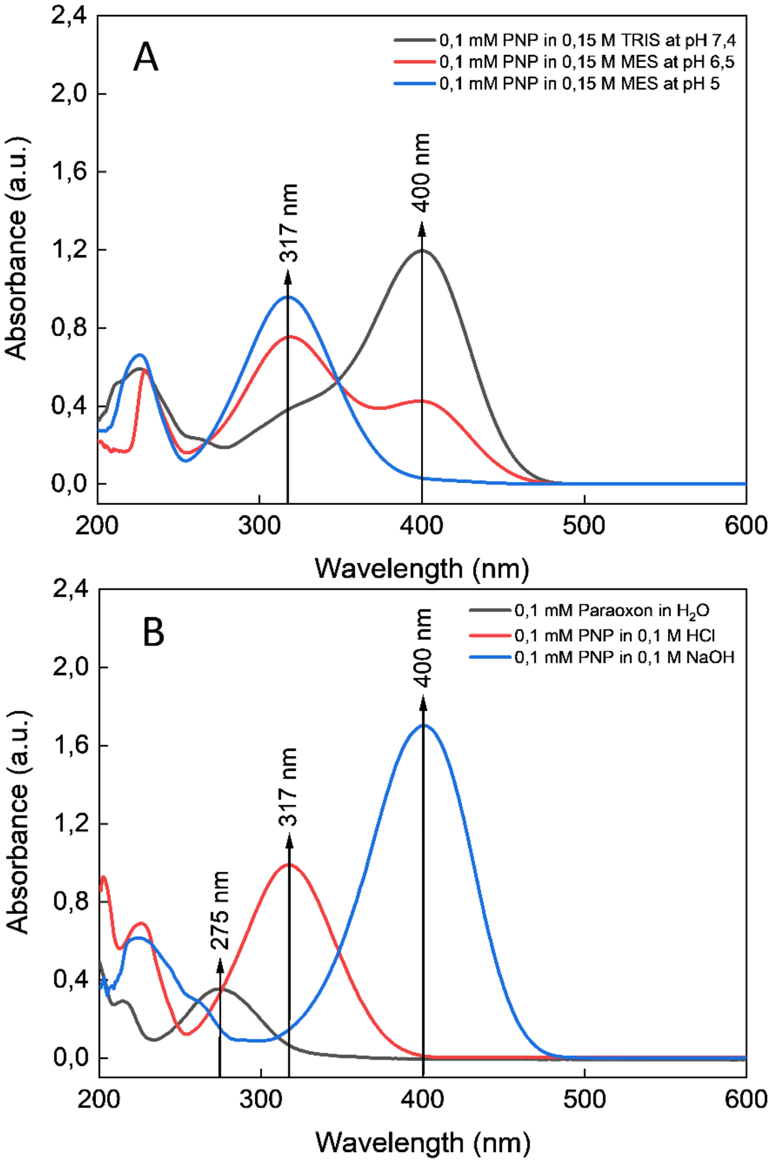

Figure S1: (A) UV-Vis spectra of 4-nitrophenol in selected buffers at various pH: 5 (MES, blue), 6.5 (MES, red) and 7.4 (TRIS, black). (B) UV-Vis spectra of Paraoxon (black), 4-nitrophenol (red) and 4-nitrophenolate (blue).

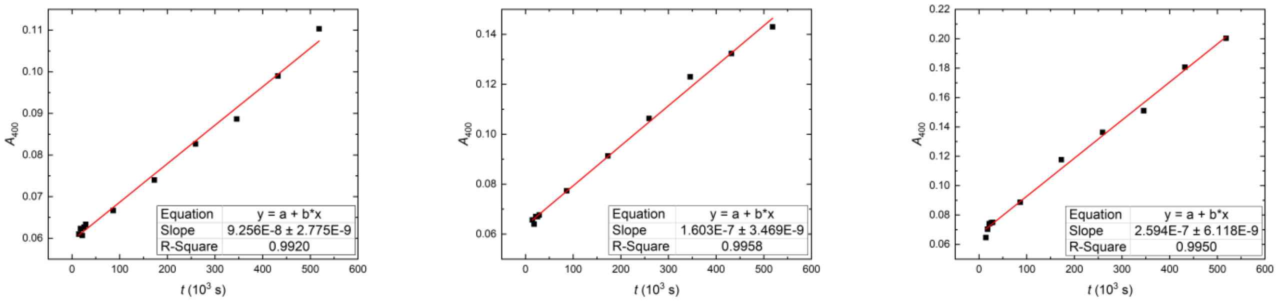

Figure S2: First-order kinetic plots of spontaneous (uncatalyzed) Paraoxon hydrolysis at various temperatures. Conditions: *c*(Paraoxon) = 1.0 mM; *c*(MES) = 0.15 M (pH 6.5), temperature (from left to right) 23, 37 and 50 °C, respectively.

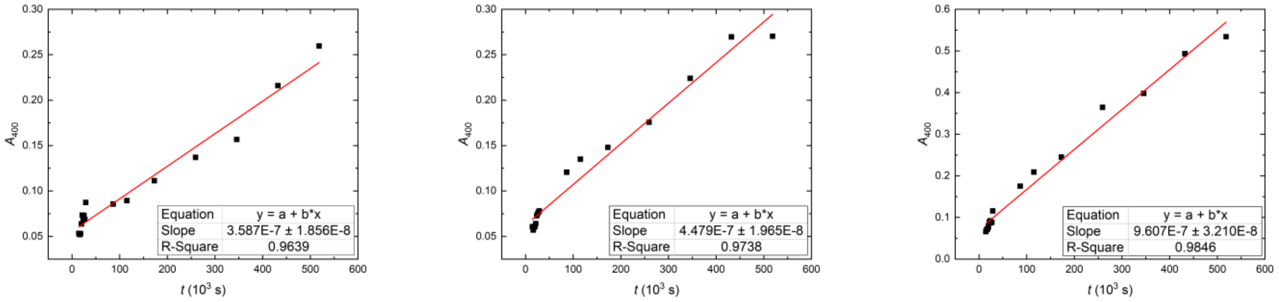

Figure S3: First-order kinetic plots of spontaneous (uncatalyzed) Paraoxon hydrolysis at various temperatures. Conditions: *c*(Paraoxon) = 1.0 mM; *c*(TRIS) = 0.15 M (pH 7.4), temperature (from left to right) 23, 37 and 50 °C, respectively.

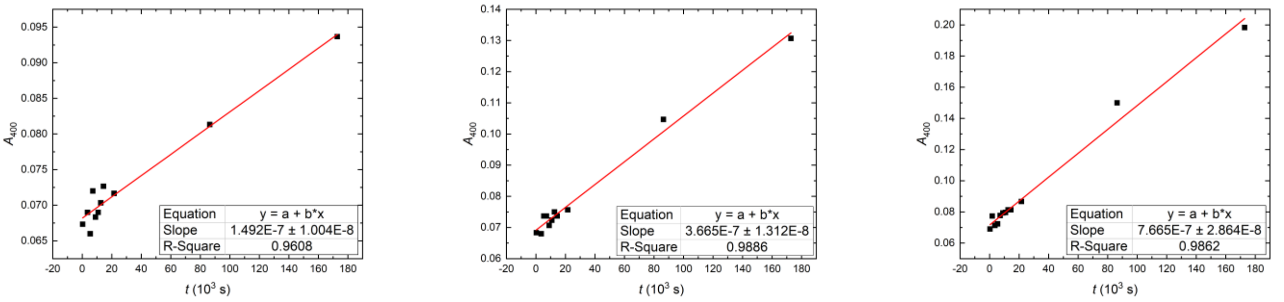

Figure S4: First-order kinetic plots of Paraoxon hydrolysis in the presence of $CuSO_4$ at various temperatures. Conditions: *c*(Paraoxon) = 1.0 mM; *c*($CuSO_4$) = 10 mM, *c*(TRIS) = 0.15 M (pH 7.4), temperature (from left to right) 23, 37 and 50 °C, respectively.

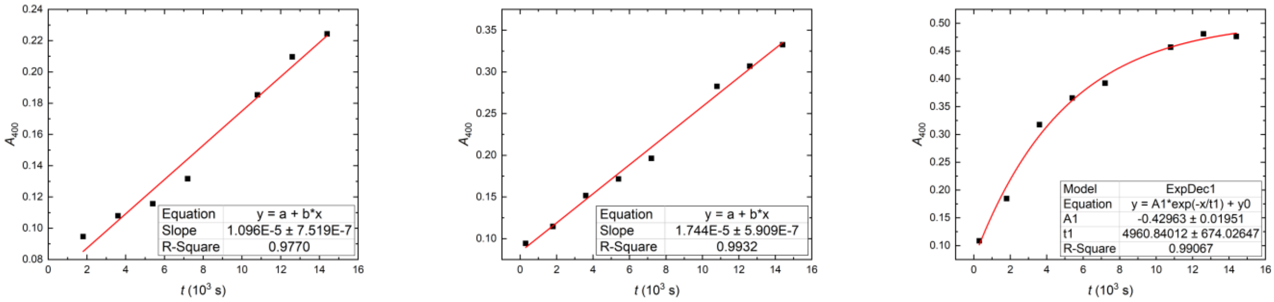

Figure S5: First-order kinetic plots of Paraoxon hydrolysis in the presence of Cu–**L1** at various temperatures. Conditions: *c*(Paraoxon) = 1.0 mᴍ; *c*(Cu–**L1**) = 10 mᴍ, *c*(TRIS) = 0.15 ᴍ (pH 7.4), temperature (from left to right) 23, 37 and 50 °C, respectively.

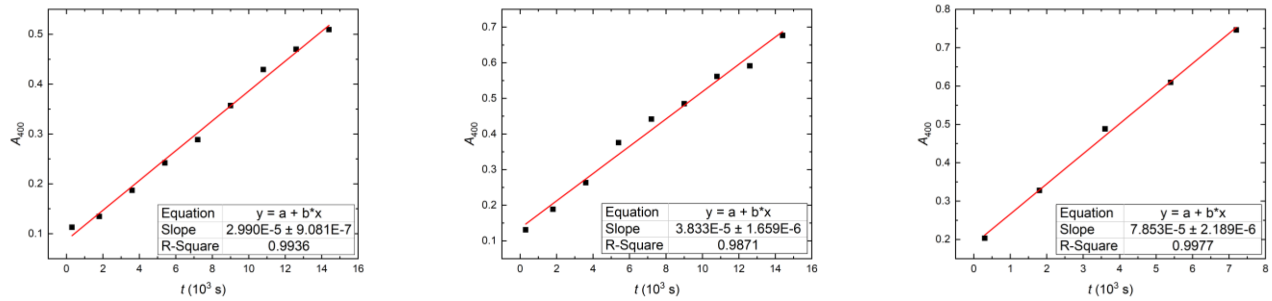

Figure S6: First-order kinetic plots of Paraoxon hydrolysis in the presence of Cu–**L2** at various temperatures. Conditions: *c*(Paraoxon) = 1.0 mᴍ; *c*(Cu–**L2**) = 10 mᴍ, *c*(MES) = 0.15 ᴍ (pH 6.5), temperature (from left to right) 23, 37 and 50 °C, respectively.

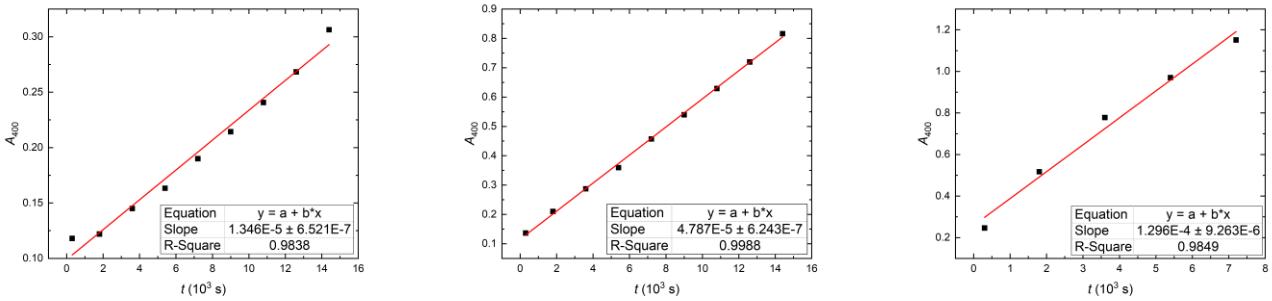

Figure S7: First-order kinetic plots of Paraoxon hydrolysis in the presence of Cu–**L2** at various temperatures. Conditions: *c*(Paraoxon) = 1.0 mᴍ; *c*(Cu–**L2**) = 10 mᴍ, *c*(TRIS) = 0.15 ᴍ (pH 7.4), temperature (from left to right) 23, 37 and 50 °C, respectively.

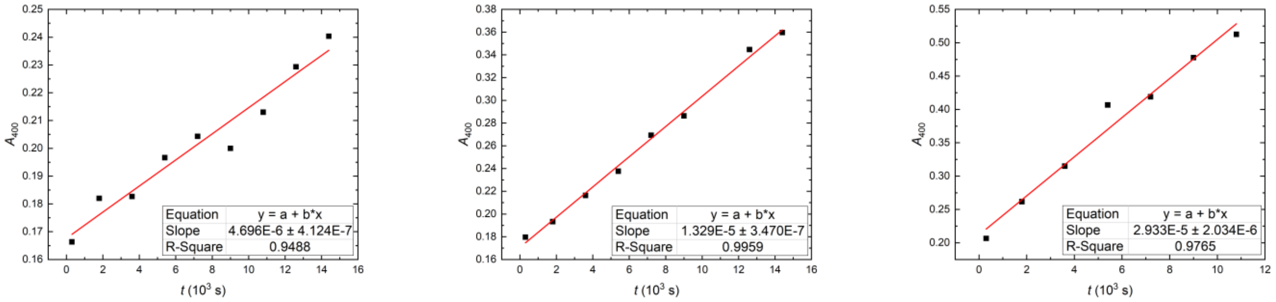

Figure S8: First-order kinetic plots of Paraoxon hydrolysis in the presence of Cu–**L2** at various temperatures in EtOH:H_2O 3:1 (v:v). Conditions: *c*(Paraoxon) = 1.0 mM; *c*(Cu–**L2**) = 10 mM, *c*(TRIS) = 0.15 M ("pH" 7.4), temperature (from left to right) 23, 37 and 50 °C, respectively.

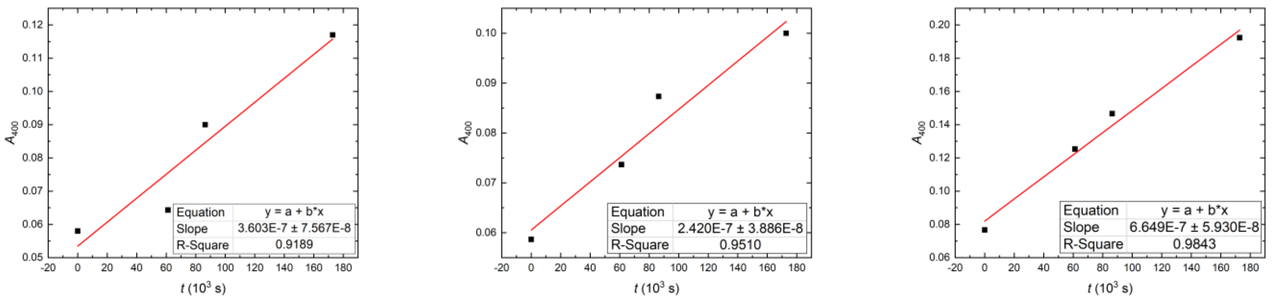

Figure S9: First-order kinetic plots of Paraoxon hydrolysis at 50 °C in the presence of (from left to right): Amberlite beads, Amberlite beads covered with metal-free 2,2'-dithiophene–**L1** co-polymer and Amberlite beads covered with 2,2'-dithiophene–**L1** co-polymer loaded with Cu(II) ions, respectively. Conditions: *c*(Paraoxon) = 1.0 mM; *m*(beads) = 30 mg, *c*(TRIS) = 0.15 M (pH 7.4), temperature 50 °C, volume of the reaction mixture 1 mL.

A
B
C

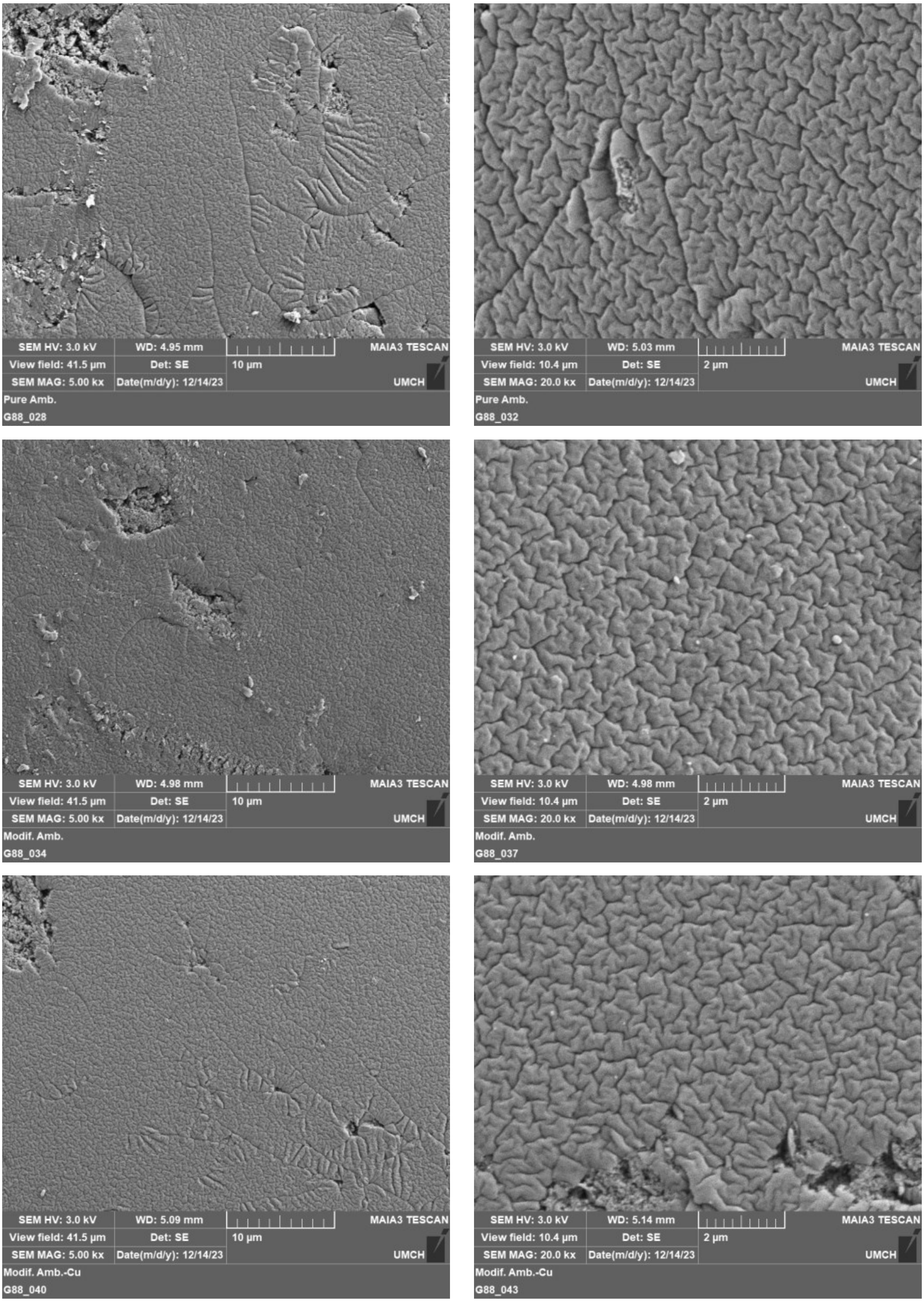

Figure S10: Morphology of materials studied by scanning electron microscopy. A) Native Amberlite beads. B) Amberlite beads covered with 2,2’-dithiophene–**L1** co-polymer. C) Final material after loading with Cu(II).

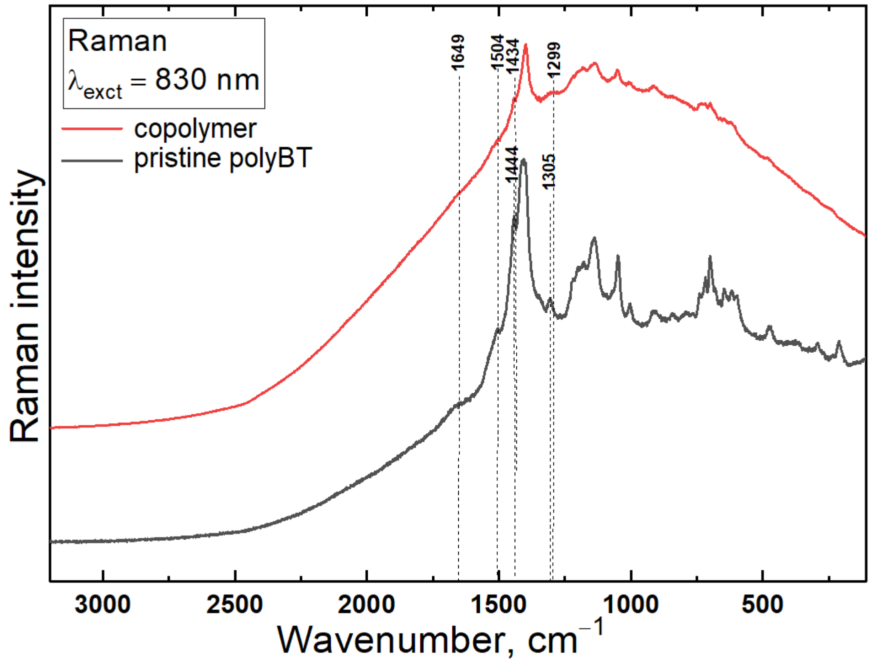

Figure S11. Raman spectra of pristine poly-2,2′-dithiophene and 2,2′-dithiophene–**L1** copolymer measured with 830 nm laser excitation.

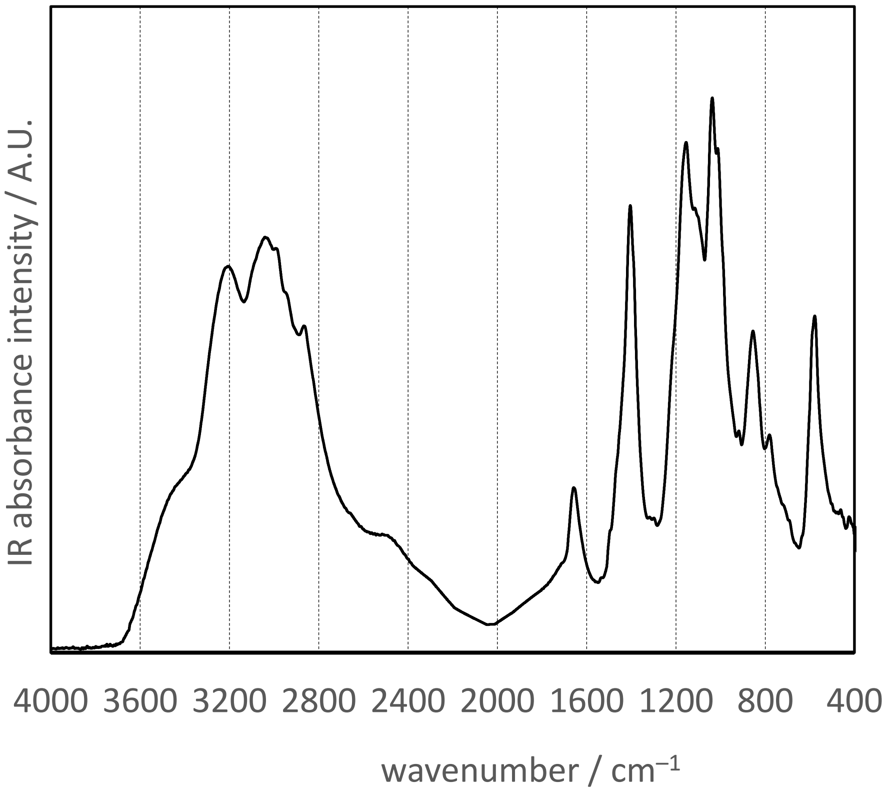

Figure S12. FTIR ATR spectrum of the 2,2′-dithiophene–**L1** copolymer. Band at 2850–2880 cm^{-1} indicates presence of aliphatic C–H bonds due to successful incorporation of L1 into the copolymer.

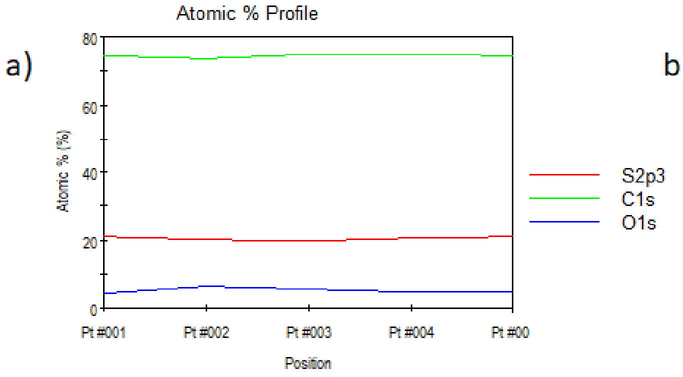

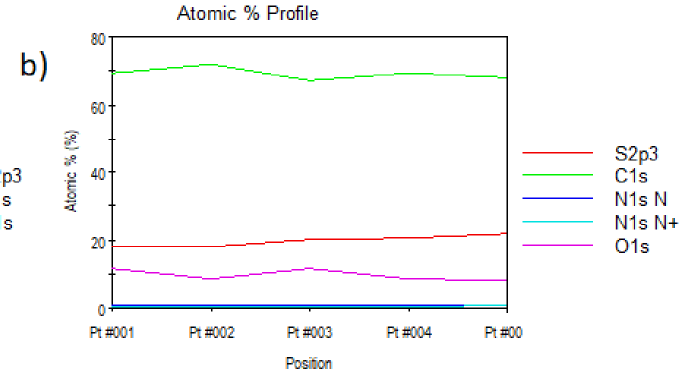

Figure S13. Graphical representations of the results of XPS measurements of a) pristine poly-2,2'-dithiophene and b) 2,2'-dithiophene–**L1** copolymer. Five different areas in the individual samples were measured. Source data are listed in Table S1.

Table S1. Compilation of XPS results (Atomic %) obtained for pristine poly-2,2'-dithiophene and 2,2'-dithiophene–**L1** copolymer. Five different areas in the individual samples were measured.

pristine poly-2.2'-dithiophene					
atom/orbital	sample 1	sample 2	sample 3	sample 4	sample 5
S2p3	21.1495	20.0058	19.838	20.4728	20.9493
C1s	74.5181	73.3673	74.727	74.6528	74.3025
O1s	4.33241	6.62687	5.43502	4.87444	4.74814

2.2'-dithiophene–**L1** copolymer					
atom/orbital	sample 1	sample 2	sample 3	sample 4	sample 5
S2p3	18.0892	17.9351	20.0409	20.838	21.9588
C1s	69.0679	71.6147	67.1985	69.0517	68.0765
N1s N	0.791331	1.02867	0.836626	1.05083	0.987052
N1s N+	0.606294	0.620065	0.525804	0.535162	0.746058
O1s	11.4453	8.80146	11.3982	8.52434	8.23168
